# Overexpression of a New Zinc Finger Protein Transcription Factor *OsCTZFP8* Improves Cold Tolerance in Rice

**DOI:** 10.1155/2018/5480617

**Published:** 2018-05-23

**Authors:** Yong-Mei Jin, Rihua Piao, Yong-Feng Yan, Mojun Chen, Ling Wang, Hongxia He, Xiaoxiao Liu, Xing-Ai Gao, Wenzhu Jiang, Xiu-Feng Lin

**Affiliations:** ^1^Jilin Academy of Agricultural Sciences, Changchun, China; ^2^Jilin University, Changchun, China; ^3^Jilin Agricultural University, Changchun, China

## Abstract

Cold stress is one of the most important abiotic stresses in rice. C_2_H_2_ zinc finger proteins play important roles in response to abiotic stresses in plants. In the present study, we isolated and functionally characterized a new C_2_H_2_ zinc finger protein transcription factor *OsCTZFP8* in rice. *OsCTZFP8* encodes a C_2_H_2_ zinc finger protein, which contains a typical zinc finger motif, as well as a potential nuclear localization signal (NLS) and a leucine-rich region (L-box). Expression of *OsCTZFP8* was differentially induced by several abiotic stresses and was strongly induced by cold stress. Subcellular localization assay and yeast one-hybrid analysis revealed that OsCTZFP8 was a nuclear protein and has transactivation activity. To characterize the function of *OsCTZFP8* in rice, the full-length cDNA of *OsCTZFP8* was isolated and transgenic rice with overexpression of *OsCTZFP8* driven by the maize ubiquitin promoter was generated using *Agrobacterium*-mediated transformation. Among 46 independent transgenic lines, 6 single-copy homozygous overexpressing lines were selected by Southern blot analysis and Basta resistance segregation assay in both T_1_ and T_2_ generations. Transgenic rice overexpressing *OsCTZFP8* exhibited cold tolerant phenotypes with significantly higher pollen fertilities and seed setting rates than nontransgenic control plants. In addition, yield per plant of *OsCTZFP8*-expressing lines was significantly (*p* < 0.01) higher than that of nontransgenic control plants under cold treatments. These results demonstrate that *OsCTZFP8* was a C_2_H_2_ zinc finger transcription factor that plays an important role in cold tolerance in rice.

## 1. Introduction

Rice (*Oryza sativa* L.) is the most important stable food crop that feeds more than two billion people worldwide. It is a warm season plant that is sensitive to cold, and its growth and production are severely affected by low temperatures [1]. Cold stress inhibits rice growth during all growth stages, ranging from vegetative to reproductive stages. During early vegetative stages, cold stress severely inhibits rice growth, such as seedling growth retardation, plant height, and tiller number reduction [1, 2]. During the reproductive stage, cold stress causes delayed heading, incomplete panicle exertion, pollen sterility, poor grain filling, reduction of seed setting rates, and finally causes yield reduction [3–7].

Plants have evolved efficient mechanisms to tolerate low temperature stress. The cold tolerance genes can be divided into three kinds according to the low temperature signal transduction pathway, including protein kinase genes, transcription factors, and functional genes. ICE-CBF-COR pathway plays an important role in plant resistance to cold stress [8–11]. A large number of genes called cold-responsive gene (COR) can be induced by cold stress. The C-repeat-binding factor (CBF) proteins regulate the COR gene expression by binding to C-repeat/dehydration-responsive (CRT/DRE) element. ICE is a bHLH (basic helix-loop-helix) transcriptional activator, which can specifically bind to a specific CBF promoter sequence at low temperatures and induces the expression of CBF genes, thus improving the cold resistance [12]. At present, there are three *CBF* genes related to chilling injury in *Arabidopsis*, including *CBF1*, *CBF2*, and *CBF3* [13]. In addition, many scientists have isolated CBF-like genes [14] in rice. Wang et al. [15] found that *OsDREB1F* transcription factors in rice could specifically bind to *cis*-acting element CRT/DRE and were induced by low temperature, salt, drought, and ABA. Ito et al. [16] showed that *OsDREB1* transgenic rice plants had obvious improvement in the characteristics of low temperature, salt resistance, and drought resistance. Chen et al. [17] found that the expression of *OsDREBL* gene was enhanced at low temperature. Nakamura [18] showed that the expression of *OsICE1* and *OsICE2* was enhanced at low temperature, and also *OsDREB1B*, *OsHsfA3* (rice heat shock factor A3), and *OsTPP1* rice genes were expressed in its transgenic rice.

Zinc finger proteins play a crucial role in resisting environmental stresses in various plants [7, 19, 20]. Based on the sequence characteristics of the conserved domain of zinc finger proteins, they can be divided into six groups: C_2_H_2_, C_3_H, C_2_C_2_, A_20_/AN_1_, C_3_H_2_C_3_, and C_3_HC_4_. The C_2_H_2_-type zinc finger protein family (also called TFIIIA-type) is one of the largest families of eukaryotic transcription factors, which have one to four finger motif(s) within each molecule and also contain a conserved QALGGH sequence within their zinc finger domain [21]. C_2_H_2_ zinc finger proteins can function as a key transcriptional regulator involved in regulating various developmental processes or responses to abiotic stresses [22]. Overexpression of the tomato C_2_H_2_ zinc finger protein transcription factor, *SlCZFP1*, confers enhanced cold tolerance in transgenic *Arabidopsis* and rice [7]. GsZFP1, a new C_2_H_2_-type zinc finger protein, is a positive regulator of plant tolerance to cold and drought stress, and *GsZFP1*-overexpressing *Arabidopsis* resulted in a greater tolerance to cold and drought stress [23]. Rice zinc finger protein ZFP245 [24] and C_2_H_2_ zinc finger protein ZFP182 [8, 9] were induced by various abiotic stresses, and overexpression of these proteins significantly enhanced multiple abiotic stress tolerances, including salt, cold, and drought tolerances in transgenic rice. Overexpression of rice zinc finger protein OsCOIN increased tolerance to chilling, salt, and drought stress and also enhanced proline level in rice [25]. Therefore, transcription factors are powerful tools for genetic engineering, as their overexpression can lead to upregulation of an array of genes under their control [1].

Rice plants are more sensitive to cold stress at the booting stages than at the seedling stages [4, 5]. Discovery of genes affecting cold tolerance at the booting stage will be helpful for developing cold tolerance cultivars and improve grain yield. Cold tolerance segregating populations are usually used for mapping and cloning genes for cold tolerance. Leucine-rich repeat receptor-like kinase CTB4a which conferred cold tolerance at the booting stage was mapped and cloned [26]. Saito et al. mapped and cloned the QTL *Ctb1* encoding an F-box protein which contributed to normal anther development under cold stress [27]. Zhou et al. [28] fine mapped *qCTB7* for cold tolerance at the booting stage on rice chromosome 7 using a near-isogenic rice. Kuroki et al. [29] mapped *qCTB8* for cold tolerance at the booting stage on rice chromosome 8.

Pollen fertilities are an important indicator of cold resistance evaluation at the reproductive stage and can be determined after cold-water treatments by obtaining pollen from spikelets and staining with 1% iodine-potassium iodide (I_2_-KI) [26, 30]. In general, the seed setting rates of plants stressed by naturally low temperatures or artificially controlled low temperatures are the most important index to evaluate the cold tolerance at the booting stage in rice [26]. In addition, physiological and biochemical indices such as osmolytes and chlorophyll, reduced reactive oxygen species, and malondialdehyde are also utilized as cold tolerance indicators [1]. In many genetic studies, genetic variation of rice varieties and agronomic trait-related correlation analysis are utilized to evaluate cold tolerance [3, 31].

Although many studies have suggested the involvement of C_2_H_2_ zinc finger protein in abiotic stress responses, their precise biological function and molecular mechanism remain to be further elucidated. In the present study, we report on the identification of OsCTZFP8, a novel zinc finger protein transcription factor in rice. We found that *OsCTZFP8* expression was induced by cold stress, and its overexpression in transgenic rice enhanced cold tolerance during the reproductive stage. Our results indicated that *OsCTZFP8* might have an important role in cold tolerance in rice.

## 2. Materials and Methods

### 2.1. Plant Materials

In the present study, Japonica rice variety, Kitaake, was used as an *Agrobacterium*-mediated transformation recipient variety. It was provided by Rice Research Institute of Jilin Academy of Agricultural Sciences.

### 2.2. Phylogenetic Analysis

Multiple sequence alignment was performed using the National Center for Biotechnology Information (NCBI) constraint-based multiple alignment tool (https://www.ncbi.nlm.nih.gov/tools/cobalt/cobalt.cgi) with the full-length amino acid sequence of OsCTZFP8 as a query. A phylogenetic tree was constructed with the aligned plant C_2_H_2_ zinc finger proteins using MEGA version 4.0 via the neighbor-joining method. The numbers at each node represent the bootstrap percentage for 1000 replicates.

### 2.3. Abiotic Stress Treatments and Spatial Expression

To investigate the expression of *OsCTZFP8* in response to various abiotic stresses, 2-week-old rice seedlings grown in 1/2 Murashige & Skoog (MS) medium [32] plates were treated with 5 *μ*M abscisic acid (ABA), 250 mM NaCl, and cold (4°C under dim light) for different time points (0, 1, 2, 5, 10, and 24 h).

For spatial expression, different organs (leaf, stem, root, flower, and endosperm) of wild type rice (Kitaake) under normal conditions were sampled for quantitative real-time PCR.

### 2.4. Quantitative Real-Time Polymerase Chain Reaction (qRT-PCR) Analysis

For qRT-PCR analysis, total RNA was isolated from abiotic stress-treated rice shoots and roots using a MiniBEST Universal RNA Extraction Kit (Takara Bio Inc., Kusatsu, Japan) according to the manufacturer's instructions. One microgram of each RNA sample was reverse transcribed to cDNA using a PrimeScript™ RT Reagent Kit with gDNA Eraser (Takara Bio Inc., Kusatsu, Japan). qRT-PCR was performed with an ABI7500HT instrument (Applied Biosystems, Foster City, USA) using FastStart Universal SYBR Green Master (Roche, Mannheim, Germany). The elongation factor1*α* (*eEF1α*) gene was used as the internal control. The sequences for gene-specific primers and internal control primers were as follows: *CTZFP8*-qF, 5′-ACGAGCCACCGGTTCAAG-3′; *CTZFP8*-qR, 5′-ATTACGCGGTGAGAAGGCGA-3′; *eEF1α*-F, 5′-TTTCACTCTTGGTGTGAAGCAGAT-3′; and *eEF1α*-R, 5′-GACTTCCTTCACGATTTCATCGTAA-3′. All experiments were performed with two biological replicates and three technical replicates for each sample. The relative quantitation method (*ΔΔ*C_T_) was applied to evaluate the quantitative variation among replicates [33, 34].

### 2.5. Subcellular Localization

The OsCTZFP8 cDNA with the stop codon removed was fused in-frame to the smGFP reporter gene, constructing CaMV35S::OsCTZFP8-smGFP vector. Transient expression assays were carried out according to the protocol described [35]. Both CaMV35S::OsCTZFP8-smGFP and the CaMV35S:: smGFP (control plasmid) were introduced into onion epidermal cells by particle bombardment. The transformed cells were cultured on MS medium at 23°C in the dark for about 20 hrs and examined under a confocal laser scanning microscope (Zeiss LSM510).

### 2.6. Transactivation Activity Assays

The transcription activity of OsCTZFP8 was examined by yeast one-hybrid assay using deletion mutants. cDNAs corresponding to FULL (225 amino acids), NTR (N-terminal region of 1–118 amino acids), and CTR (C-terminal region of 119–225 amino acids) were inserted into the *EcoR*I and *Pst*I sites of pGBKT7 (Clontech, USA) fusing with the GAL4 DNA-binding domain. The constructed yeast expression vectors, pGBKT7-OsCTZFP8-FULL, pGBKT7-OsCTZFP8-NTR, and pGBKT7-OsCTZFP8-CTR, empty vector of pGBK7 as a negative control, were transferred to yeast AH109 strain, respectively. The yeast-transformed strains were placed on the SD/−Trp or SD/−Trp + X-*α*-gal medium placed at 30°C for 2~3 days.

### 2.7. P_Ubi_:*OsCTZFP8* Plant Expression Vector Construction and Rice Transformation

Total RNA was isolated from 2-week-old rice seedlings and reverse transcribed with oligo (dT) primer using SuperScript III reverse transcriptase (Invitrogen, Carlsbad, USA). Full-length open reading frames of *OsCTZFP8* were amplified by PCR with the synthesized first-strand cDNA using forward primer 5′-TTTAACTGCAGATGGCGATGGCATTTTTGG-3′ (*Pst*I site underlined) and reverse primer 5′-TTTAAGTCGACCGTGCAGCTGCTGAATTAC-3′(*Sal*I site underlined, stop codon deleted) and inserted into the *Pst*I and *Sal*I sites of the p3300-Ubi vector under the control of the maize ubiquitin (Ubi) promoter and selection marker of the phosphinothricin acetyltransferase (*bar*) gene. The constructed plant expression vector P_Ubi_:*OsCTZFP8* was introduced into Japonica rice (*Oryza sativa* “Kitaake”) by *A. tumefaciens*- (EHA105-) mediated transformation. The callus culture and transformation procedures were based on methods described by Hiei and Komari [36] with minor modifications. Scutellum-derived embryonic calli were cocultured with EHA105 containing the P_Ubi_:*OsCTZFP8* for 3 days and then transferred to the selection medium containing 30 mg/L Basta (Bayer, Leverkusen, Germany). After 3-4 selection times, resistant calli were regenerated and developed into transformed plantlets.

PCR amplification was performed with T_0_ independent transgenic plants and *bar* gene-specific primers: Bar-F, 5′-GCACCATCGTCAACCACTACATCGAG-3′ and Bar-R, 5′-TGAAGTCCAGCTGCCAGAAACCCAC-3′.

The protein expression of the *bar* gene was detected using T_0_ transgenic rice plants and nontransgenic (NT) control plants by a LibertyLink strip detection kit (EnviroLogix, Portland, USA) according to the manufacturer's instructions.

### 2.8. Selection of Homozygous *OsCTZFP8* Transgenic Plants with Single-Copy Insertion

To determine the stable integration of the *OsCTZFP8* gene, Southern blot analyses were performed with transgenic lines. Plant genomic DNA was extracted by the cetyl trimethyl ammonium bromide (CTAB) method [37]. Approximately 40 *μ*g of genomic DNA was digested with restriction enzyme *Hind*III (Takara Bio Inc., Kusatsu, Japan). The digested DNA fragments were separated on 0.8% agarose gel and transferred to a Hybond N^+^ nylon membrane (Amersham Biosciences, Buckinghamshire, UK). Southern blot probe was PCR amplified using Ubi promoter specific forward primer (Ubi-SF, 5′-TTTAGCCCTGCCTTCATACG-3′) and OsCTZFP8 gene-specific reverse primer (ZFP8-SR, 5′-ATTACGCGGTGAGAAGGCGA-3′), and then the PCR products were labeled with DIG-High Prime DNA Labeling and Detection Starter Kit II (Roche, Mannheim, USA). After hybridizing with the DIG-labeled Ubi-*OsCTZFP8* probe, the membrane was treated with CSPD and exposed to X-ray film.

The Basta resistance seed germination assay was performed to select single-copy homozygous lines based on the procedures described by Jin et al. [38] with minor modifications. A total of 150 seeds of each individual line were sterilized and placed on 1/2 MS medium containing 30 mg/L Basta under long-day conditions at 28°C. Germinated seeds were determined as Basta resistant if they had a radicle length of 1.5 mm on the 5th day after seeding. The lines that were demonstrated as being 3 : 1 Mendelian segregation of Basta resistance in T_1_ generation and 100% resistance in T_2_ generation were selected as single-copy homozygous lines.

### 2.9. Evaluation of Cold Tolerance

T_2_ generation of *OsCTZFP8*-overexpressing lines and NT plants were grown at the cold tolerance evaluation nursery in Jilin Province, China. Germinated seeds of rice were sown on April 25, 2017, in a plastic-film house and transplanted into the cold tolerance evaluation nursery on June 2, 2017. Each line was transplanted at a planting density of 27 cm between rows and 12.5 cm between plants. A mixed commercial fertilizer was applied at the ratio of 140-80-80 kg/hm^2^ of N-P-K.

The cold treatments were conducted in a cold-water irrigation nursery where the water temperature was artificially controlled. Cold water was obtained from aquifers cooled by electrical cooling systems and monitored by a temperature sensing system. The water was set to a temperature of 19°C with a 15 cm water depth from young panicle formation stage to booting stage for 30 days. Normal conditional treatments were conducted in a natural temperature water irrigation nursery. The water obtained from aquifers was stored in a water tank for several days to maintain natural temperatures (daily mean temperature of 22.5~28.5°C from 5 July to 5 August) and then irrigated into the rice with a 10 cm water depth.

Pollen fertilities and seed setting rates were evaluated as cold evaluation indices under cold treatments compared to normal conditional treatments. Pollen fertilities were determined by anthesis staining with 1% I_2_-KI solution, following the method described by Shinjyo [39]. The pollen was collected from spikelets and pounded on a slide, stained with I_2_-KI solution, and observed under an Olympus BX43 microscope (Olympus, Tokyo, Japan). The numbers of blue-stained pollen grains and total grains were counted to determine pollen fertilities. Seed setting rates were measured after harvest by counting the shrunken grains and full grains. There were five panicles per plant, ten plants per line were measured, and the average was calculated.

### 2.10. Agronomic Trait Measurements

Agronomic traits of *OsCTZFP8*-overexpressing lines and NT control plants were investigated under cold treatments and normal conditions. Agronomic traits measured were tillers per plant, panicle length, 1000-grain weight, and yield per plant. Five plants for each repetition were measured, and the average was obtained from three replicates.

## 3. Results

### 3.1. Isolation and Sequence Analyses of OsCTZFP8

**Figure 1:**
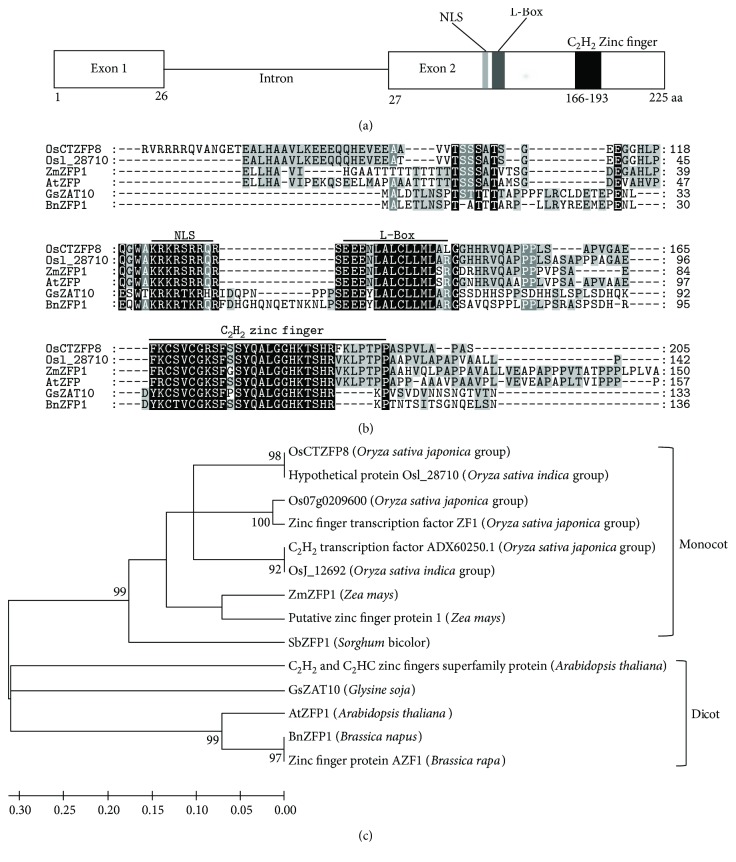
Analyses of OsCTZFP8 protein architecture and its relationship to other zinc finger protein in plants. (a) Schematic representation of the 225 amino acids of OsCTZFP8. Two exons, one intron, NLS, L-box, and C_2_H_2_ zinc finger domain are indicated. (b) Amino acid sequence alignment of rice OsCTZFP8 with zinc finger proteins from rice Osl_28710 (*Oryza sativa* L., gi|34015350), maize ZmZFP1 (*Zea mays*, gi|242032883), *Arabidopsis* AtZFP1 (*Arabidopsis thaliana*, gi|15240742), soybean GsZAT10 (*Glycine soja*, gi|734330588), and winter oilseed rape ZFP1 (*Brassica napus*, gi|685279228) was performed using a NCBI constraint-based multiple alignment tool and visualized with GeneDoc version 2.7. One zinc finger motif, putative NLS, and L-Box are indicated. Identical and conserved amino acids are displayed in black and grey backgrounds, respectively. (c) The phylogenetic tree of OsCTZFP8 homologs in plants. Phylogenetic analysis was conducted by MEGA version 4.0 using fourteen OsCTZFP8 homologs selected from multiple sequence alignment. A neighbor-joining tree was built using the bootstrap method with 1000 replicates. The numbers at each node represent the bootstrap percentage. In addition to the members cited in (b), the following protein sequences were incorporated into the analysis: rice *Os07g0209600* (*Oryza sativa japonica*, gi|937925668), rice *OsZF1* (*Oryza sativa japonica*, gi|34393438), rice C_2_H_2_ transcription factor (*Oryza sativa japonica*, gi|323388891), rice OsJ_12692 (*Oryza sativa indica*, gi|125588016), maize putative zinc-finger protein1 (*Zea mays*, gi|195640880), *Sorghum SbZFP1* (*Sorghum bicolor*, gi|670405684), *Arabidopsis* C_2_H_2_ and C_2_HC zinc fingers superfamily protein (*Arabidopsis thaliana* C_2_H_2_ and C_2_HC zinc fingers superfamily protein, gi|15229643), and *Brassica BrAZF1* (*Brassica rapa*, gi|923538325).

In our previous work, we mapped a quantitative trait locus for cold tolerance during the reproductive stage on rice chromosome 8 and delimited it to a 99.4 kb region (our unpublished data). Fourteen putative genes were located in this region, and only LOC_Os08g20580 was considered to be the cold tolerance candidate gene by bioinformatics analyses. LOC_Os08g20580 was predicted to be encoded with a zinc finger protein transcription factor and then was designated as OsCTZFP8 (*Oryza sativa* cold tolerance zinc finger protein in chromosome 8) by us. OsCTZFP8 is 225 amino acids long and contains one C_2_H_2_ zinc finger domain with a plant-specific QALGGH motif as well as a conserved leucine-rich motif (L-box) and contains a putative nuclear localization signal (NLS) with a consensus sequence of KRKRSRR (Figures 1(a) and 1(b)). Comparisons of the amino acid sequences between OsCTZFP8 and other C_2_H_2_ zinc finger protein homologs in plants (*O. sativa*, *Zea mays*, *Sorghum bicolor*, and *Arabidopsis thaliana*) showed that the C_2_H_2_ zinc finger domains were highly conservative in these plant species (Figure 1(b)). To investigate the evolutionary relationship among plant C_2_H_2_ zinc finger proteins involved in stress responses, a phylogenetic tree was constructed with full-length amino acid sequences. The results showed that OsCTZFP8 was clustered on the same branch with nine C_2_H_2_ zinc finger proteins from monocotyledonous plants, which distinguished them from dicotyledonous species (Figure 1(c)). Promoter analysis of the 2 kb *OsCTZFP8* promoter region using the PLACE software program (http://www.dna.affrc.go.jp/PLACE/signalscan.html) found that it contained a low-temperature responsiveness (LTR) *cis*-acting element and abscisic acid responsive element (ABRE). It was also predicted to respond to abiotic stimulus by the Chinese National Rice Data Center (http://www.ricedata.cn) and the Rice Genome Annotation Project funded by the National Science Foundation (http://rice.plantbiology.msu.edu). Based on these observations, we speculated that OsCTZFP8 might confer abiotic stress tolerance in rice.

### 3.2. Transcriptional Expression of *OsCTZFP8*

**Figure 2:**
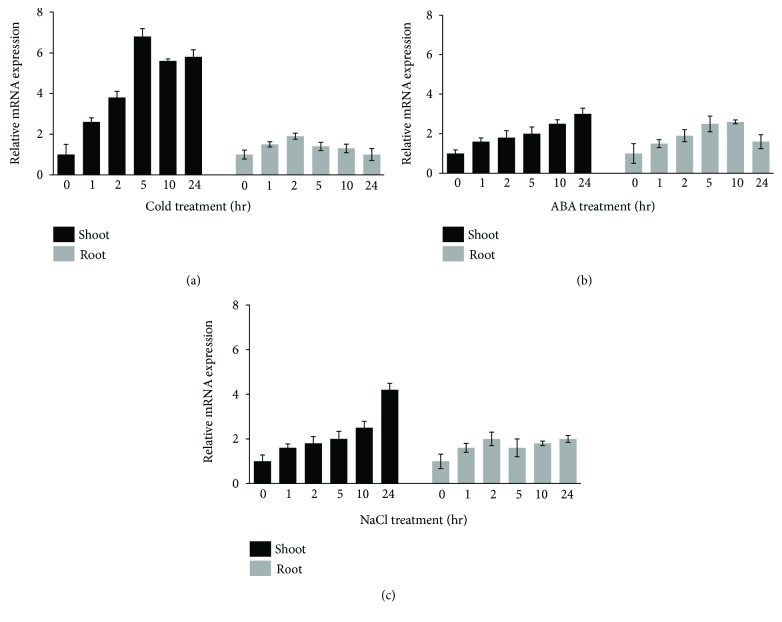
Expression patterns of *OsCTZFP8* transcript in response to abiotic stresses. qRT-PCR was performed with 2-week-old NT plants exposed to cold (4°C), ABA (5 *μ*M), and NaCl (250 mM) treatments at different time points. The expression of *eEF1α* was used as an internal control. Data present the means ± SE of two biological replicates.

To investigate the possible involvement of *OsCTZFP8* in environmental stress responses, qRT-PCR was performed with 2-week-old rice plants exposed to cold, ABA, and NaCl treatments at different time points. As shown in Figure 2, transcription of *OsCTZFP8* was induced by all three stresses; however, the induction showed different kinetic patterns among shoots and roots. In shoots, the transcription level of *OsCTZFP8* was significantly elevated under cold and NaCl treatments, whereas it was induced weakly under ABA treatments: *OsCTZFP8* was induced by cold and reached a maximum (~6-fold) at 5 h and induced ~4-fold within 24 h under NaCl treatment; however, it was only increased up to ~3-fold in 24 h under ABA treatment. In roots, *OsCTZFP8* was induced weakly under ABA, NaCl, and cold treatments compared to the NT group (Figure 2). These results indicated that *OsCTZFP8* was involved in plant responses to cold and salinity stresses in rice. We promptly attempted to investigate the biological roles of *OsCTZFP8* in response to cold stress.

**Figure 3:**
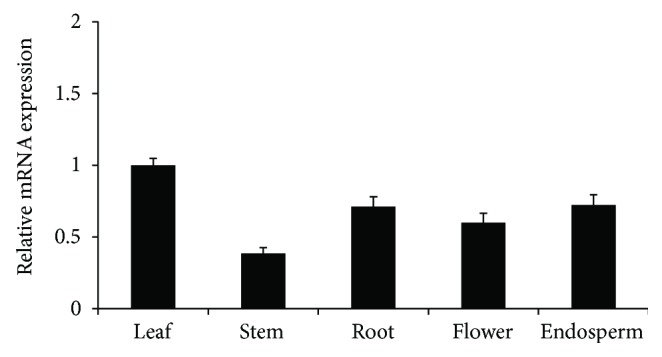
Spatial expression pattern of *OsCTZFP8*. qRT-PCR was performed with total RNA isolated from different organs of wild-type rice under normal conditions. The expression of *eEF1α* was used as an internal control. Data present the means ± SE of two biological replicates.

To examine the spatial expression of *OsCTZFP8*, qRT-PCR was performed with total RNA isolated from different organs of wild-type rice. As shown in Figure 3, transcription of *OsCTZFP8* was differentially expressed in different rice organs. It was expressed the most abundantly in the leaf followed by root, endosperm, flower, and stem.

### 3.3. OsCTZFP8 Is a Nuclear Protein and Has Transactivation Activity

**Figure 4:**
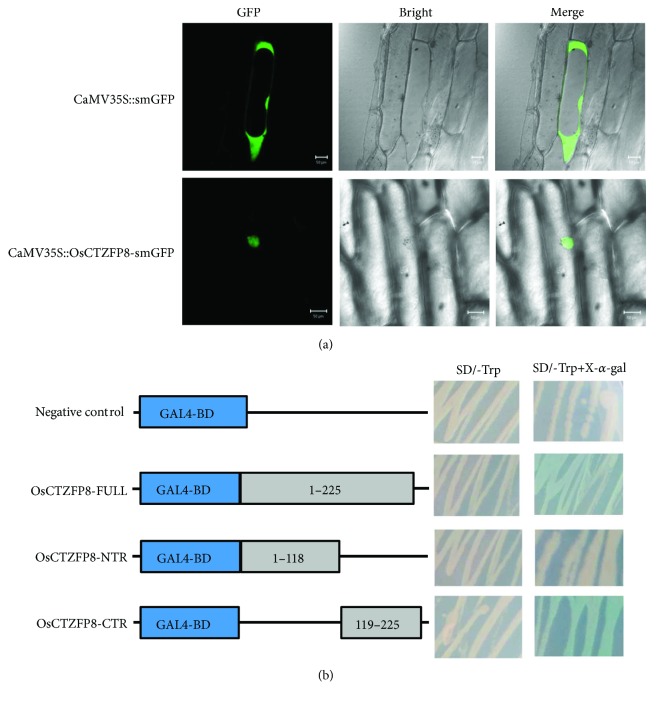
Subcellular localization and transactivation activity of OsCTZFP8. (a) Subcellular localization of OsCTZFP8. Bar represents 50 *μ*m. (b) Transactivation activity of OsCTZFP8 in yeast. Deletion mutants of OsCTZFP8 are illustrated on the left and transactivation activity on the right. Empty pGBKT7 was used as the negative control.

The deduced OsCTZFP8 protein contains a stretch of basic residues KRKRSRR, which may function as a potential NLS. To determine the subcellular localization of OsCTZFP8, OsCTZFP8 was fused to the 5′ end of the soluble-modified green fluorescent protein (smGFP) gene under the control of the CaMV 35S promoter and introduced into onion epidermal cells by particle bombardment. As shown in Figure 4(a), the fusion protein, CaMV35S::OsCTZFP8-smGFP, was localized predominantly to the nucleus of onion cells, whereas CaMV35S::smGFP alone was distributed in both the cytoplasm and the nucleus. These results indicate that OsCTZFP8 was a nuclear protein.

To examine the transactivation activity of OsCTZFP8, a yeast one-hybrid system was used [40]. FULL, NTR, and CTR of OsCTZFP8 were fused to the GAL4 DNA-binding domain and then cotransformed with the *Lac*Z reporter into yeast cells. The results showed that FULL and CTR of OsCTZFP8 displayed transactivation activity; however, NTR had no transactivation activity (Figure 4(b)). The results suggest that OsCTZFP8 was a transcriptional activator.

### 3.4. Generation of *OsCTZFP8-*Overexpressing Transgenic Rice

**Figure 5:**
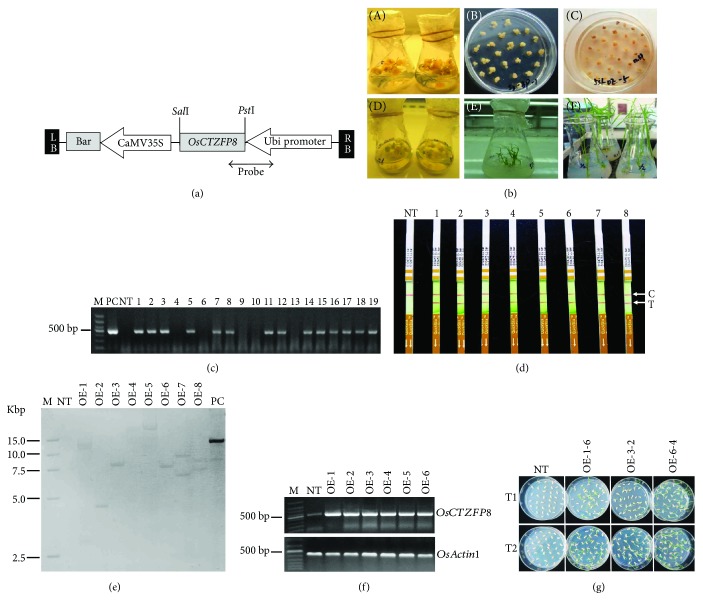
Generation and identification of *OsCTZFP8*-overexpressing rice. (a) Schematic representation of the T-DNA region on the P_Ubi_:*OsCTZFP8* construct. The construct contains the *bar* gene and a maize ubiquitin (Ubi) promoter. The small double arrow bar represents the Ubi*-OsCTZFP8* probe used for Southern blot analysis. (b) *Agrobacterium*-mediated rice seed transformation. The procedures included induction of callus (A), subculture (B), cocultivation and Basta screening (C), differentiation (D), rooting (E), and planet acclimatization (F). (c) PCR analyses for T_0_ transgenic rice using *bar* gene-specific primers. PC: plasmid DNA; NT: nontransgenic control plant; 1~19: T_0_ generation of independent transgenic rice. (d) LibertyLink strip detections of T_0_ transgenic rice plants; NT: nontransgenic control plant; 1~10: T_0_ transgenic plants; C: control line; T: test line. (e) Southern blot analysis of *OsCTZFP8*-overexpressing lines. *Hind*III-digested genomic DNA from T_1_ generation was separated on agarose gel and hybridized with DIG-labeled Ubi*-OsCTZFP8* probe; NT: nontransgenic control plants; OE-1~OE-8: overexpressing lines; PC: plasmid DNA. (f) mRNA expression of *OsCTZFP8* in overexpressing lines. One-step RT-PCR was performed using total RNA extracted from 2-week-old rice leaves. The expression of *OsActin1* was used as an internal control; NT: nontransgenic control plants; OE-1~OE-6: single-copy insertion overexpressing lines. (g) Basta resistance segregation assay. Seeds were germinated on 1/2 MS medium (30 mg/L Basta), and Basta resistance were determined at the 5th day. The experiments were performed in two repetitions; NT: nontransgenic control plants; OE-1~6 and OE-3-2: overexpressing lines.

To elucidate whether the biological function of the *OsCTZFP8* gene confers cold tolerance, transgenic rice plants carrying P_Ubi_:*OsCTZFP8*, in which *OsCTZFP8* was driven by the maize Ubi promoter, were generated. The full-length open reading frame of *OsCTZFP8* cDNA was cloned into the plant expression vector p3300-Ubi (Figure 5(a)) and transformed into rice by *Agrobacterium-*mediated transformation. A total of 46 transformed rice plants with Basta resistance were obtained via callus induction, subculture, *Agrobacterium* infection, Basta resistance selection, shoot differentiation, root induction, and plant transplantation procedures (Figure 5(b)). Among them, 36 plants were confirmed to be positive plants by *bar* gene-specific PCR detection and LibertyLink strip analysis of bar protein (Figures 5(c) and 5(d)). These results indicated that the transgene was integrated into the rice genome and successfully expressed in the protein level.

Subsequently, to obtain single-copy insertion transgenic lines, Southern blot analysis was performed. After hybridizing with Ubi*-OsCTZFP8*-specific probe (Figure 5(a)), six independent single-copy lines were obtained from T_1_* OsCTZFP8* transgenic rice (Figure 5(e)). To check the transcripts of *OsCTZFP8*, RT-PCR was performed using single-copy lines with *OsCTZFP8*-specific primers. The results showed that *OsCTZFP8* transcript accumulated at higher levels in *OsCTZFP8* transgenic lines than in NT control plants (Figure 5(f)). Using Basta resistance segregation analyses, two single-copy homozygous lines (OE-1-6 and OE-3-2), which exhibited one-locus Mendelian segregation ratio of 3 : 1 in T_1_ generation and 100% for Basta resistance in T_2_ generation, were selected for further study (Figure 5(g)).

### 3.5. Overexpression of *OsCTZFP8* Improves Cold Tolerance of Transgenic Rice

**Figure 6:**
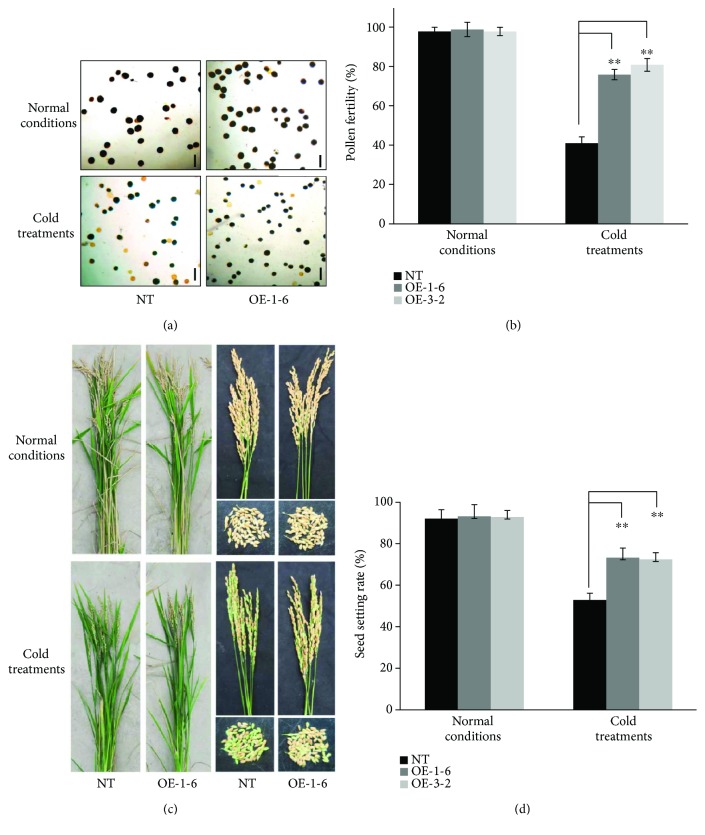
Cold tolerance evaluation of *OsCTZFP8*-overexpressing lines and NT control plants. (a) Pollen fertilities were determined using a 1% iodine-potassium iodide (I_2_-KI) solution after cold treatments. The pollen was picked up from spikelets and pounded on the slide, stained with the I_2_-KI, and observed under an Olympus microscope; NT: nontransgenic control plants; OE-1-6: *OsCTZFP8*-overexpressing line. Scale bars represented 100 *μ*m. (b) Blue-stained pollen grains were counted to determine pollen fertilities; NT: nontransgenic control plants; OE-1-6 and OE-3-2: *OsCTZFP8*-overexpressing lines. Data represent the means ± SE (*n* = 5) of three independent experiments. ^∗∗^Significantly different from the NT at *p* < 0.01. (c) Seed setting rates were observed at harvest. Photographs were taken for the upper part of the ground of rice plants, panicles, and the seeds of overexpressing lines and NT; NT: nontransgenic control plants; OE-1-6, overexpressing line. (d) Seed setting rates were measured after harvest by counting the shrunken grains and full grains. Five panicles per plant and ten plants per line were measured, and the average was calculated. Data represent the means ± SE (*n* = 5) of two independent experiments. ^∗∗^Significantly different from the NT at *p* < 0.01; NT: nontransgenic control plants; OE-1-6 and OE-3-2: *OsCTZFP8*-overexpressing lines.

To examine whether *OsCTZFP8-*overexpressing rice confers for cold tolerance, cold treatments were performed using *OsCTZFP8-*overexpressing lines and NT control plants. Then, pollen fertilities of *OsCTZFP8-*overexpressing lines and NT control plants were determined by I_2_-KI staining. Under normal conditions, all pollen grains of *OsCTZFP8-*overexpressing line (OE-1-6 and OE-3-2) and NT control plants were stained dark blue with no significant differences between them, exhibiting approximately 98% of pollen fertilities. Under cold treatments, more than three quarters of the pollen in OE-1-6 and OE-3-2 were stained dark blue, which explained 76~81% of fertility, whereas less than half the pollen of NT control plants were stained dark blue and others were stained reddish brown, resulting in only approximately 41% of fertilities (Figures 6(a) and 6(b)). These results indicate that pollen fertilities were significantly improved in Os*CTZFP8*-overexpressing lines than in NT control plants under cold treatments.

Seed setting rates are the most important index for cold tolerance evaluation at the reproductive stage. Seed setting rates of *OsCTZFP8*-overexpressing lines and NT control plants were tested under normal conditions and cold treatments by counting the shrunken grains and full grains after harvest. Under normal conditions, OE-1-6, OE-3-2, and NT showed almost the same level of seed setting rates (>92%). However, under cold treatments, seed setting rates of OE-1-6 and OE-3-2 were significantly higher than those of NT control plants (*p* < 0.01): for example, OE-1-6 and OE-3-2 showed 73.3% and 72.5% seed setting rates, respectively, while NT control plants showed only 52.9% seed setting rates (Figures 6(c) and 6(d)). These results demonstrate that pollen fertilities and seed setting rates were higher in *OsCTZFP8*-overexpressing lines than in NT control plants under cold treatments during the reproductive stage, suggesting *OsCTZFP8* overexpression could improve cold tolerance in rice.

### 3.6. Agronomic Traits of *OsCTZFP8-*Overexpressing Transgenic Rice

**Figure 7:**
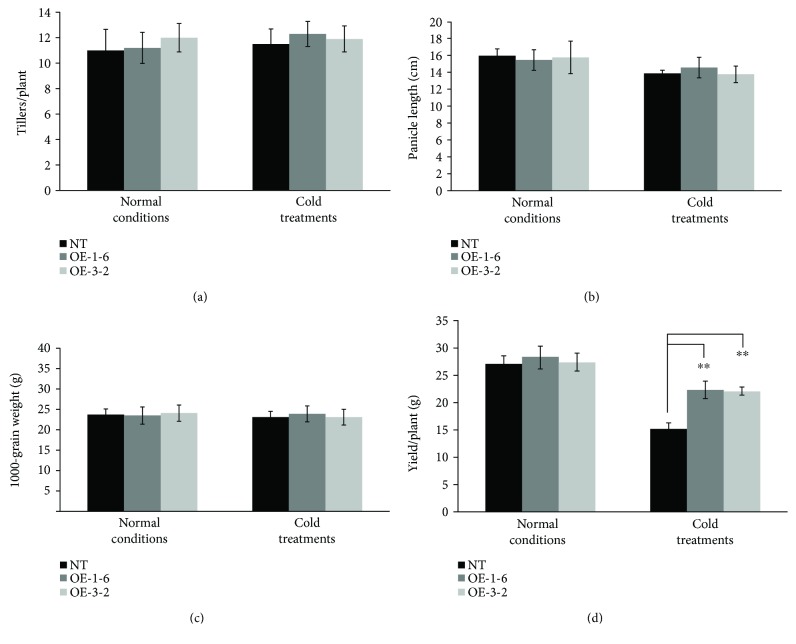
Agronomic traits of *OsCTZFP8*-overexpressing lines and NT control plants. Agronomic traits including tillers per plant, panicle length, 1000-grain weight, and yield per plant were measured under normal conditions and cold treatments. Five plants for each repetition were measured and the average was taken from three replicates ^∗∗^ Significantly different from the NT at *p* < 0.01.

Agronomic traits of *OsCTZFP8*-overexpressing lines and NT control plants were evaluated under normal conditions and cold treatments. Under normal conditions, all agronomic traits tested had no differences between *OsCTZFP8*-overexpressing lines and NT control plants. Under cold treatments, tillers per plant, panicle length, and 1000-grain weight had no differences between *OsCTZFP8*-overexpressing lines and NT control plants; however, yield per plant of *OsCTZFP8*-expressing lines was significantly (*p* < 0.01) higher than that of NT control plants (Figure 7).

## 4. Discussion

The C_2_H_2_ zinc finger transcription factors are usually thought to be involved in plant development and have various adaptive responses to environmental stress [22]. In the present study, we characterized a new C_2_H_2_ zinc finger transcription factor *OsCTZFP8* from rice. Sequence analyses revealed that OsCTZFP8 had high identity with other C_2_H_2_ zinc finger proteins and shared one zinc finger motif containing a conserved plant-specific QALGGH amino acid sequences (Figure 1(b)), which has been proven to be critical for DNA-binding activity [41]. In addition, the C-terminus of the *OsCTZFP8* gene contains a typical L-box (Figure 1(b)), which plays roles in protein interactions or maintaining the folded structure [42]. Multiple sequence alignment and phylogenetic analysis showed that OsCTZFP8 was clustered on the same branch as nine C_2_H_2_ zinc finger proteins from monocotyledonous plants, which distinguished it from dicotyledonous species (Figure 1(c)). This indicated that dicotyledonous plants and monocotyledon plants have differences in genetic characteristics of C_2_H_2_ zinc finger proteins and probably play different regulation roles in plant responses to abiotic stresses. Subcellular localization assay and yeast one-hybrid analysis (Figure 4) revealed that OsCTZFP8 was a nuclear protein and has transcriptional activation activity, implying that *OsCTZFP8* was a transcription factor.

The promoter region of *OsCTZFP8* contains various abiotic stress-responsive *cis*-acting elements including LTR and ABRE, suggesting that *OsCTZFP8* might be upregulated by interacting with its upstream genes binding to these *cis*-elements. Accordingly, transcript expression analysis revealed that *OsCTZFP8* was differentially induced by several stresses such as cold, ABA, and high salinity, particularly, strongly elevated in response to cold stress. These results suggest that *OsCTZFP8* might be involved in cold stress responses in rice (Figure 2).

To gain more knowledge regarding the function of *OsCTZFP8*, full-length coding sequence (CDS) of *OsCTZFP8* from rice was cloned by reverse transcription and *OsCTZFP8*-overexpressing rice driven by the maize Ubi promoter was constructed by *Agrobacterium*-mediated transformation (Figures 5(a) and 5(b)). The *OsCTZFP8* gene was stably integrated into the rice genome as confirmed by PCR and Southern blot analysis using T_0_ transgenic plants (Figures 5(c) and 5(e)). In addition, single-copy insertion lines were selected by Southern blot analysis to maintain stability of *OsCTZFP8* inheritance and convenience of homozygous selection (Figure 5(e)). Selection of single-copy insertion transgenic plants is important for transgenic breeding, because multiple gene copies can lead to instability of expression and inheritance of the transgene even gene silencing [43, 44]. Transcription levels of *OsCTZFP*8 were accumulated higher in single-copy insertion overexpressing lines than in NT rice (Figure 5(f)), indicating that the *OsCTZFP8* gene was overexpressed successfully and gene silencing did not occur in transcription levels. When using Basta resistance segregation analyses, two homozygous lines from single-copy insertion transgenic lines were finally selected for further analysis (Figure 5(g)). Homozygous lines can stably maintain consistency of genetic and phenotypic characters and accelerate the generation process and reduce workload [38].

To effectively and correctly evaluate cold tolerance, factors such as cold stress temperature and duration of stress exposure, phase of the developmental stage, and phenotypic indices must be considered when defining a treatment method. When choosing a cold-water temperature for tolerance evaluations, it is important to consider that low temperatures allow for the identification of the highest levels of tolerance and high temperatures help identify moderate tolerance. It has been reported that cold-water temperature was determined ranging from 15 to 19°C with a depth of 5 to 20 cm for different cold tolerance evaluation purposes [29, 45]. In our cold-water treatment facility, the temperature of the cold water was controlled reliably because cold water was obtained from aquifers and cooled by an electrical cooling system based on the water temperature sensing system in the nursery. Under cold treatments, pollen fertilities of *OsCTZFP8*-overexpressing lines were significantly (*p* < 0.01) higher than NT control plants (Figure 6). Consistent with this, seed setting rates of *OsCTZFP8*-overexpressing lines were significantly higher than those of the NT control plants (*p* < 0.01) under cold treatments (Figure 6). In addition, the yield per plant of *OsCTZFP8*-overexpressing lines was significantly (*p* < 0.01) higher than that of NT control plants (Figure 7). All these results confirmed that *OsCTZFP8*-overexpressing lines increased tolerance to cold stress during the reproductive stage, indicating that *OsCTZFP8* plays a role in cold stress response.

Transcription factor is a critical component of the gene regulatory networks that plays a central role in response to abiotic stress. It regulates expression of a number of stress-responsive genes to cope with stress [46, 47]. To better understand the molecular role of *OsCTZFP8* in cold tolerance responses, it would be useful to identify downstream target genes and interaction proteins by transcriptome sequencing and yeast two-hybrid system as well as their expression changes in *OsCTZFP8*-overexpressing plants compared with NT control plants.

In summary, the present study identified a new zinc finger transcription factor OsCTZFP8 in rice that can be induced by various abiotic stresses. Overexpression of *OsCTZFP8* in rice can improve cold tolerance during the reproductive stage by enhancing pollen fertilities and seed setting rates as well as yield per plant, suggesting a promising utility of this gene in genetic improvement of cold tolerance. The molecular regulation mechanism of *OsCTZFP8* for cold tolerance and other abiotic stress tolerance could be further elucidated in the future studies.

## Data Availability

The data used to support the findings of this study are available from the corresponding author upon request.
